# Sarcopenia supersedes subjective global assessment as a predictor of survival in colorectal cancer

**DOI:** 10.1371/journal.pone.0218761

**Published:** 2019-06-20

**Authors:** Pankaj G. Vashi, Kimberly Gorsuch, Li Wan, Danielle Hill, Christel Block, Digant Gupta

**Affiliations:** Cancer Treatment Centers of America (CTCA) at Midwestern Regional Medical Center, Zion, IL, United States of America; National Cancer Center, JAPAN

## Abstract

**Background:**

Sarcopenia, the presence of skeletal muscle mass depletion, can be objectively quantified, whereas subjective global assessment (SGA) is a widely utilized subjective instrument to assess nutritional status. Both the presence of sarcopenia and SGA-assessed malnutrition, in isolation, have been shown to be associated with worse overall survival in a wide range of cancers. However, there is no research evaluating the independent prognostic significance of both the presence of sarcopenia and malnutrition as part of the same analysis. We investigated the impact of sarcopenia on overall survival in colorectal cancer specifically controlling for malnutrition.

**Methods:**

We examined a consecutive case series of 112 patients with colorectal cancer first seen at our institution between August 2012 and October 2017. Using computed tomography (CT) imaging, the cross-sectional area of muscles at the L3 vertebral level was measured and then divided by height squared to calculate skeletal muscle index (SMI). Sarcopenia was defined as SMI ≤38.5 cm^2^/m^2^ for women and ≤52.4 cm^2^/m^2^ for men. SGA assessments were completed within 2 weeks of CT imaging. The association of sarcopenia and malnutrition with overall survival was assessed using univariate and multivariate Cox regression analysis.

**Results:**

Median age at presentation was 53.3 years. Sixty-six (58.9%) patients had metastatic disease at diagnosis. Using SMI, 46 (41.1%) patients were sarcopenic, while 66 (58.9%) were non-sarcopenic. Using SGA, 69 (61.6%) patients were assessed as well-nourished, while 43 (38.4%) were malnourished. Of 69 patients classified as well-nourished by SGA, 22 (31.9%) were sarcopenic. Similarly, of 43 patients categorized as malnourished by SGA, 19 (44.2%) were non-sarcopenic. On multivariate analysis, after adjusting for age, gender, tumor stage, BMI, treatment history and SGA, patients with sarcopenia had 3 times greater risk of mortality compared to those without sarcopenia (p = 0.001). The median survival of patients with both sarcopenia and malnutrition (n = 24) was 14.6 months (95% CI: 10.5 to 18.6) compared to the median survival of 25.9 months (95% CI: 7.8 to 44.0) in patients who were either sarcopenic or malnourished but not both (n = 41; p = 0.001). The median survival of patients who were non-sarcopenic and well nourished (n = 48; p = 0.001) was 38.6 months (95% CI: 25.6 to 51.6).

**Conclusions:**

The exploratory study suggests that presence of sarcopenia supersedes the presence of malnutrition as a predictor of survival in colorectal cancer. Co-existence of sarcopenia and malnutrition is associated with worse survival in colorectal cancer compared to just one of those conditions being present. Prospective studies with large sample sizes are needed to confirm these findings.

## Introduction

Sarcopenia, the progressive loss of skeletal muscle mass and strength, has been identified as an independent predictor of several unfavorable outcomes such as physical disability, poor quality of life, longer hospital stay, injuries and death [1,2]. While there are no uniform criteria to measure and define sarcopenia to date, it is commonly classified according to sex-specific definitions based on the skeletal muscle index (SMI), which may be reproducibly measured using cross-sectional imaging modalities such as computed tomography (CT) [3]. CT scans are routinely performed in elderly individuals, those who have cancer, as well as those undergoing major surgical procedures, and the muscle metrics generated using CT have been used as biomarkers of sarcopenia in these populations [1,4].

Subjective global assessment (SGA), on the other hand, is a widely utilized subjective instrument to assess nutritional status (a large component of which is muscle mass). The SGA, a gold-standard for bedside nutrition assessment, combines data from subjective and objective aspects of medical history (weight change, dietary intake change, nutrition impact symptoms, and changes in functional capacity) and physical examination (fat loss, muscle wasting, ankle or sacral edema and ascites) [5]. Following evaluation, patients are classified into three distinct classes of nutritional status; well nourished (SGA A), moderately malnourished (SGA B) and severely malnourished (SGA C). The SGA relies on a collective clinical judgment and has been validated in a number of diverse patient populations, including cancer patients [6–11].

Both CT-assessed sarcopenia [3,12–21] and SGA-assessed malnutrition [22–28], in isolation, have been shown to be associated with worse overall survival in a wide range of cancers. Both these factors are not only important as prognostic indicators, but are also potentially modifiable. However, there is little to no research evaluating the independent prognostic significance of both sarcopenia and malnutrition as part of the same analysis and it is not clear if one factor can supersede the other in predicting overall survival in colorectal cancer. Colorectal cancer is an ideal setting to evaluate the combined associations of these factors with overall survival because of the almost universal availability of CT images either as part of diagnosis or follow-up. We therefore investigated the impact of sarcopenia on overall survival in colorectal cancer specifically controlling for the effects of malnutrition.

## Methods

### Study design and patient population

This was a retrospective study of a consecutive case series of 112 patients with colorectal cancer first seen at Cancer Treatment Centers of America (CTCA) Chicago between August 2012 and October 2017. All adult colorectal cancer patients coming to CTCA for treatment during the above time period were considered eligible irrespective of their age, gender or any other clinical or demographic characteristic. The inclusion criteria were: a diagnosis of histologically-confirmed colorectal adenocarcinoma, age greater than 18 years, having CT scans within 2 weeks (either before or after) of presenting to our hospital, and a nutritional assessment completed within two weeks of the CT scan. There only exclusion criterion was the lack of availability of CT scans completed within 2 weeks of reporting to our hospital. Patients were not excluded based on their prior treatment history. We included a consecutive case series of patients to avoid non-response and minimize the probability of selection bias. All eligible patients were identified from the hospital’s tumor registry.

The present study was conducted according to the guidelines laid down in the Declaration of Helsinki and was approved by the western institutional review board (WIRB). The need for written informed consent was waived by WIRB because there was no direct patient contact in this study. This study involved collection of existing data from patient records in such a manner that subjects cannot be identified, directly or through identifiers linked to the subjects. Patient records/information was anonymized prior to analysis.

### Sarcopenia and nutritional assessment

Skeletal muscle mass was assessed by a retrospective examination of CT scans (SliceOmatic 4.3, Tomovision, Montreal, Canada) obtained before the start of treatment. The third lumbar vertebra (L3) was set as a landmark, and two consecutive slices were selected to measure the cross-sectional areas of skeletal muscle, which were identified using Hounsfield unit thresholds of −29 to +150. Skeletal muscle at the L3 level included psoas, paraspinal muscles (erector spinae and quadratus lumborum), and abdominal wall muscles (transversus abdominus, external and internal obliques, and rectus abdominus). The mean value of the image was computed for each patient. These values were normalized against the square of the patient's height (m^2^) to obtain the skeletal muscle index (SMI, cm^2^/m^2^). Although there is no universally-accepted definition of sarcopenia, in accordance with the previously published literature [29–31], sarcopenia was defined as SMI ≤38.5 cm^2^/m^2^ for women and ≤52.4 cm^2^/m^2^ for men. These cut-offs were chosen because they have been validated and linked with impaired outcomes in gastrointestinal cancer [31].

All patients included in this study received a consultation with a dietitian before undergoing any treatment at our institution. During the nutritional consultation, the dietitian went through the SGA instrument with the patient. This was followed by a physical examination in which particular attention was paid to the signs of fat loss and muscle wasting as well as alterations in fluid balance such as presence of ankle and sacral edema and ascites. Following the consultation, the patient’s nutritional status was categorized as either well-nourished (SGA A), or moderately malnourished (SGA B), or severely malnourished (SGA C) [5]. Because SGA measures short-term nutrition impact symptoms in the previous two weeks, these assessments were completed within 2 weeks of CT imaging, as part of the inclusion criteria.

### Statistical analysis

Descriptive statistics were conducted to compare baseline measures and differences between sarcopenic and non-sarcopenic groups, using Fisher's exact test for categorical variables and Mann-Whitney U-test for continuous variables. Overall survival was the primary end point and was defined as the time interval between the date of diagnosis (for newly diagnosed patients) or the date of first contact at our institution (for previously treated patients) and the date of patient’s death from any cause or the date of last contact/last known to be alive. The presence of sarcopenia was used as the primary independent variable in this study. Other control variables investigated for their relationship with overall survival were age at presentation, gender, BMI, prior treatment history, stage at diagnosis and SGA. The prior treatment history variable categorized patients into those who had received definitive cancer treatment elsewhere before coming to CTCA and those who were newly diagnosed at CTCA. The stage at diagnosis variable was dichotomized into metastatic (stage IV) and non-metastatic disease (stages I-III). Patients were dichotomized as normally nourished vs. malnourished, collapsing moderate and severely malnourished into one nutritionally compromised group for more meaningful comparisons.

The overall survival curves were evaluated by the Kaplan-Meier method using the log-rank test. Univariate Cox proportional hazards models were used to determine which variables showed individual prognostic value for survival. Multivariate Cox proportional hazards models were then performed to evaluate the joint prognostic significance of all variables significant on univariate analysis. The effect of individual variables on patient survival was expressed as hazard ratios (HRs) with 95% confidence intervals (CIs). Cox regression with time-invariant covariates assumes that the ratio of hazards for any two groups remains constant in proportion over time. We checked this assumption by examining log-minus-log (LML) plots for categorical predictors and an extended Cox model with time-dependent covariates for continuous predictors [32]. Potential multicollinearity was assessed using tolerance and variance inflation factor (VIF) to verify that multicollinearity was not significantly influencing model coefficients. Tolerance and VIF assess multicollinearity by regressing each independent variable on all the other independent variables in the equation simultaneously. Tolerance (1-R^2^) indicates the percentage of variance in the independent variable that is not accounted for by other independent variables. VIF, which is the reciprocal of tolerance, indicates the degree to which the standard errors are inflated due to the levels of multicollinearity. Tolerance smaller than 0.25 and VIF greater than 4.0 was considered to indicate multicollinearity [33,34].

Owing to its retrospective nature, no formal sample size calculations were conducted for this study. All data were analyzed using IBM SPSS version 23.0 (IBM, Armonk, NY, USA). A difference was considered to be statistically significant if the p value was less than or equal to 0.05.

## Results

### Patient characteristics

The median age at presentation was 53.3 years. Sixty-three (56.2%) patients were males while 49 (43.8%) were females. Eighty-five (75.9%) patients had colon cancer while 27 (24.1%) had rectal cancer. Sixty-six (58.9%) patients had metastatic disease at diagnosis, whereas 2 (1.8%), 7 (6.3%) and 37 (33%) had stage I, II and III disease at diagnosis. Stages I and II were combined with stage III for the purpose of this analysis because of a very small number of patients with stage I and II disease. Sixty-eight (60.7%) patients were newly diagnosed while 44 (39.3%) were previously treated before presenting to our hospital. The median BMI was 28.6 kg/m^2^. Using SGA, 69 (61.6%) patients were well-nourished while 43 (38.4%) were malnourished. Using SMI, 46 (41.1%) patients were sarcopenic while 66 (58.9%) were non-sarcopenic. The most commonly received chemotherapies were FOLFIRINOX (fluorouracil, leucovorin, irinotecan and oxaliplatin) with bevacizumab or capecitabine (n = 15), FOLFOX (fluorouracil, leucovorin and oxaliplatin) (n = 9), FOLFIRI (fluorouracil, leucovorin and irinotecan) (n = 8), oxaliplatin and capecitabine (n = 6), FOLFIRI with bevacizumab and capecitabine (n = 4), oxaliplatin, capecitabine and bevacizumab (n = 3), FOLFIRINOX with bevacizumab (n = 3), FOLFIRINOX (n = 2), FOLFIRINOX with capecitabine (n = 2), FOLFOX with bevacizumab (n = 2), FOLFOX with capecitabine (n = 2), taxanes (n = 2), FOLFIRI with bevacizumab (n = 1) and FOLFOX with bevacizumab and capecitabine (n = 1), while 34 patients received single agents or different combinations of these drugs. The remaining 18 patients received targeted therapies such as cetuximab, panitumumab, regorafenib, trametinib and sunitinib.

**Table 1** shows the baseline characteristics of the patient population stratified by sarcopenia status (yes versus no). Patients with sarcopenia were significantly older and had a significantly lower BMI as compared to those without sarcopenia. There were no other significant associations between sarcopenia and other baseline characteristics.

**Table 1:** Baseline patient characteristics (N = 112).

Variable	Sarcopenia		P value#
	Yes (n = 46)	No (n = 66)	
Median age (years, range)	55.6 (31.1–72.8)	51.2 (35.7–74.9)	0.05*
Median BMI (kg/m^2^, range)	24.9 (15.8–34.8)	31.6 (20.9–54.5)	<0.001*
Male (%)	56.5%	56.1%	0.96
Metastatic disease (%)	65.2%	54.5%	0.26
Newly diagnosed (%)	65.2%	57.6%	0.42

#P value compares the distribution of baseline characteristics across 2 categories of sarcopenia

*P < = 0.05

*BMI* body mass index

Cross-tabulation analysis between sarcopenia and SGA (**Table 2**) revealed that of 69 patients classified as well-nourished by SGA, 22 (31.9%) were sarcopenic. Similarly, of 43 patients classified as malnourished by SGA, 19 (44.2%) were non-sarcopenic.

**Table 2:** Cross-tabulation analysis of baseline sarcopenia and SGA (N = 112).

Baseline sarcopenia	Baseline SGA		Total	P value
	Well-nourished	Malnourished		
**No**	47 (68.1%)	19 (44.2%)	66	0.01*
**Yes**	22 (31.9%)	24 (55.8%)	46	
Total	69	43	112	

*P < = 0.05

Numbers in parentheses are column percentages. *SGA* subjective global assessment.

### Univariate analysis—predictors of overall survival

**Fig 1:**
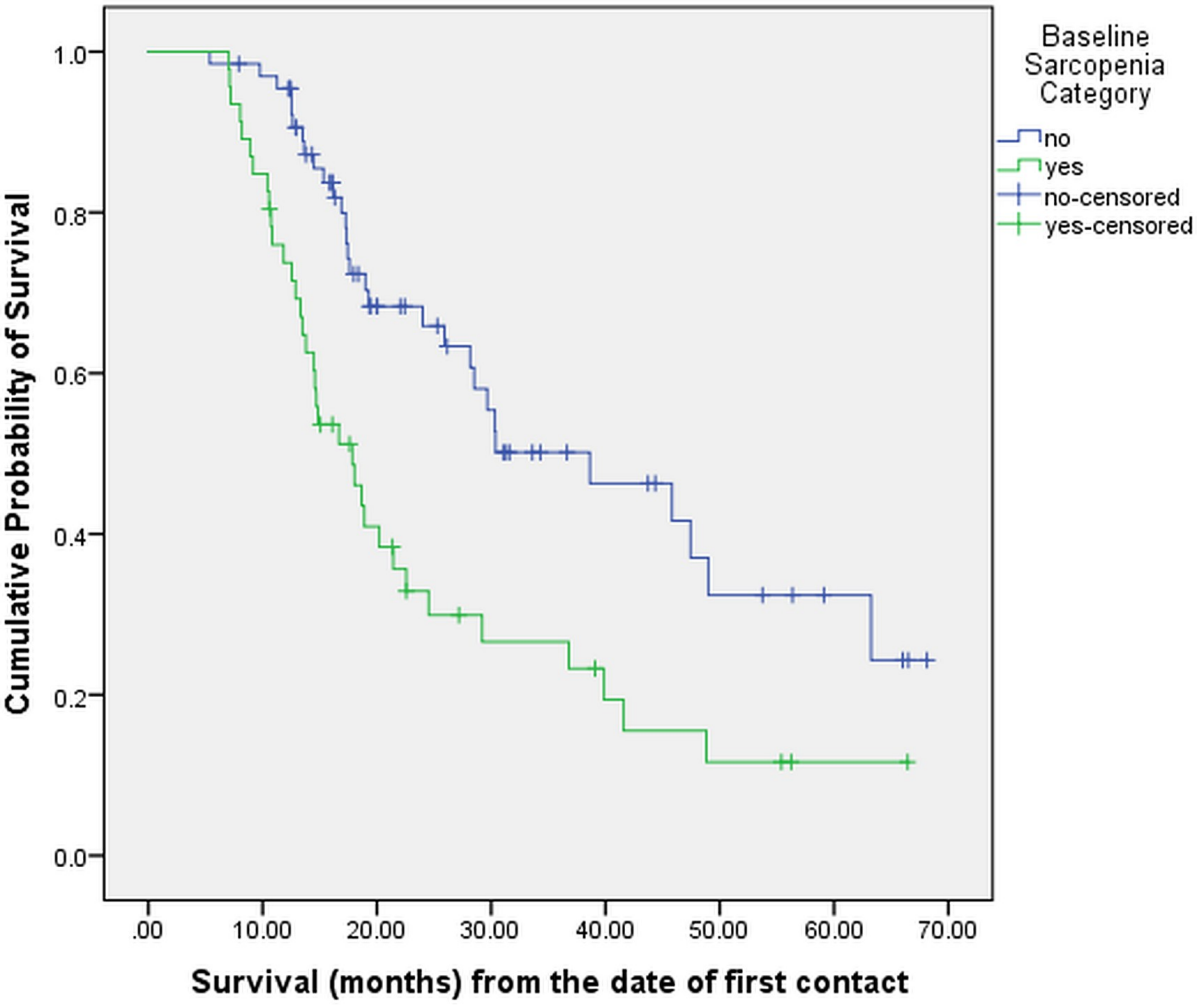
Overall survival stratified by baseline sarcopenia (N = 112).

**Fig 2:**
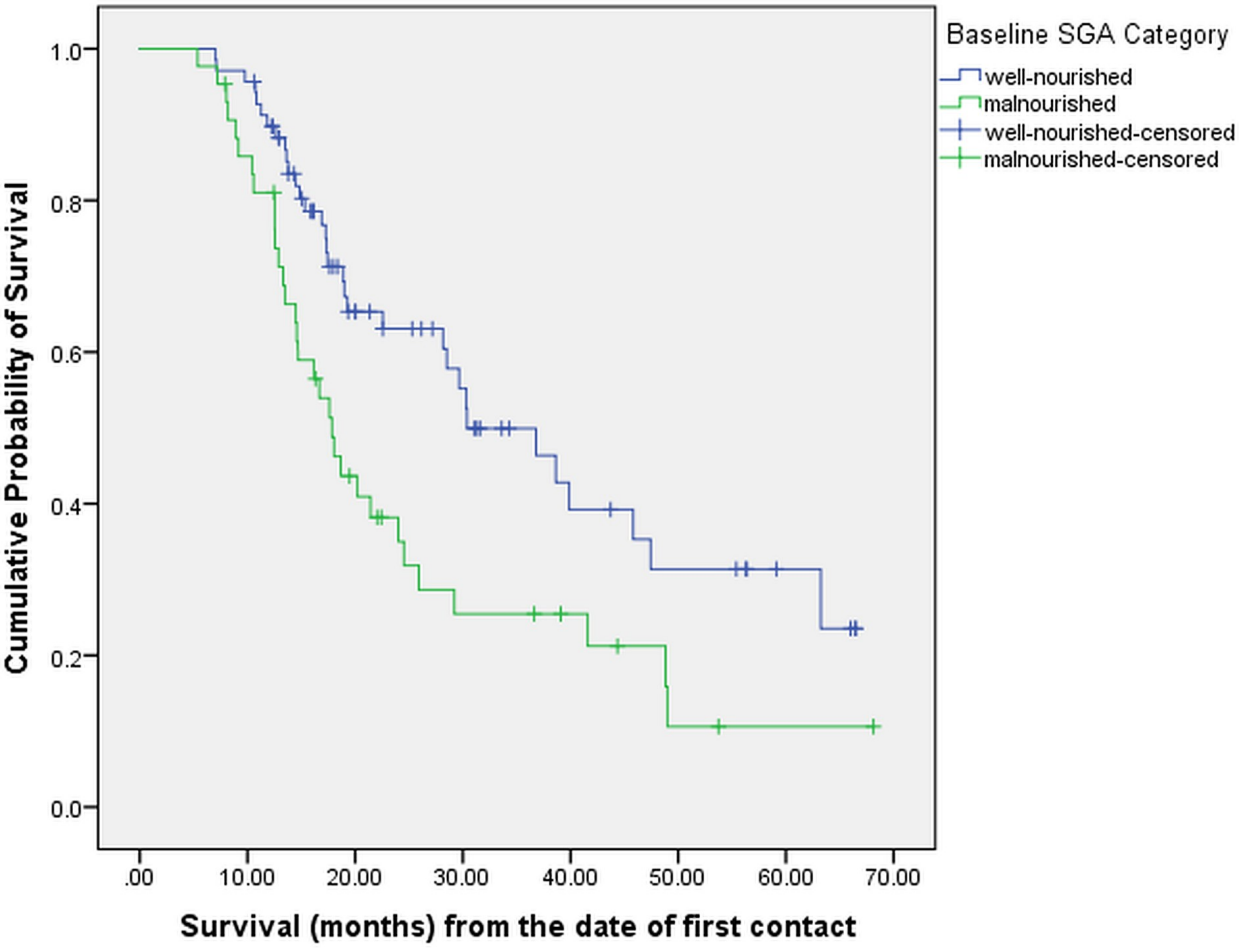
Overall survival stratified by baseline SGA (N = 112).

At the time of this analysis (November 2018), 65 (58%) patients had expired while 47 (42%) were considered censored. On Kaplan-Meier analysis, the median overall survival for the entire patient cohort was 25.9 months (95% CI: 17.6–34.2 months). On univariate analysis (**Table 3**), sarcopenia and malnutrition were statistically significantly associated with worse survival. The median survival in sarcopenic and non-sarcopenic patients was 17.8 and 38.6 months respectively (p = 0.001), as shown in **Fig 1**. Similarly, the median survival in malnourished and well-nourished patients was 17.8 and 30.4 months respectively (p = 0.006), as shown in **Fig 2**. Age, gender, BMI, treatment history and stage at diagnosis were not significantly associated with survival.

**Table 3:** Univariate survival analysis (N = 112).

Categorical Variables	Median Survival in Months	95% CI	P value
Sarcopenia			0.001*
No (n = 66)	38.6	20.6–56.7	
Yes (n = 46)	17.8	13.1–22.6	
SGA			0.006*
Well-nourished (n = 69)	30.4	20.0–40.7	
Malnourished (n = 43)	17.8	14.8–20.8	
Gender			0.57
Male (n = 63)	25.9	13.7–38.1	
Female (n = 49)	24.5	12.8–36.3	
Prior treatment history			0.46
Newly-diagnosed (n = 68)	22.6	11.9–33.1	
Previously-treated (n = 44)	29.7	21.1–38.2	
Stage at Diagnosis			0.17
Non-metastatic (n = 46)	39.9	10.9–68.9	
Metastatic (n = 66)	24.0	16.6–31.4	
**Continuous Variables**	**HR**	**95% CI**	**P value**
Age (years)	0.99	0.97–1.03	0.83
BMI (kg/m^2^)	0.98	0.95–1.03	0.51

*P < = 0.05

*SGA* subjective global assessment, *BMI* body mass index, *HR* hazard ratio, *CI* confidence interval

When the analysis was conducted separately for stages I-III and stage IV, the following results were obtained. For stages I-III patients (n = 46), the median survival in sarcopenic and non-sarcopenic patients was 39.8 (95% CI: 5.7–74.0) and 45.8 (95% CI: 6.5–85.2) months respectively (p = 0.40); whereas for stage IV patients (n = 66), the median survival in sarcopenic and non-sarcopenic patients was 14.7 (95% CI: 10.3–19.1) and 30.3 (95% CI: 12.2–48.4) months respectively (p = <0.001).

### Multivariate analysis—predictors of overall survival

**Fig 3:**
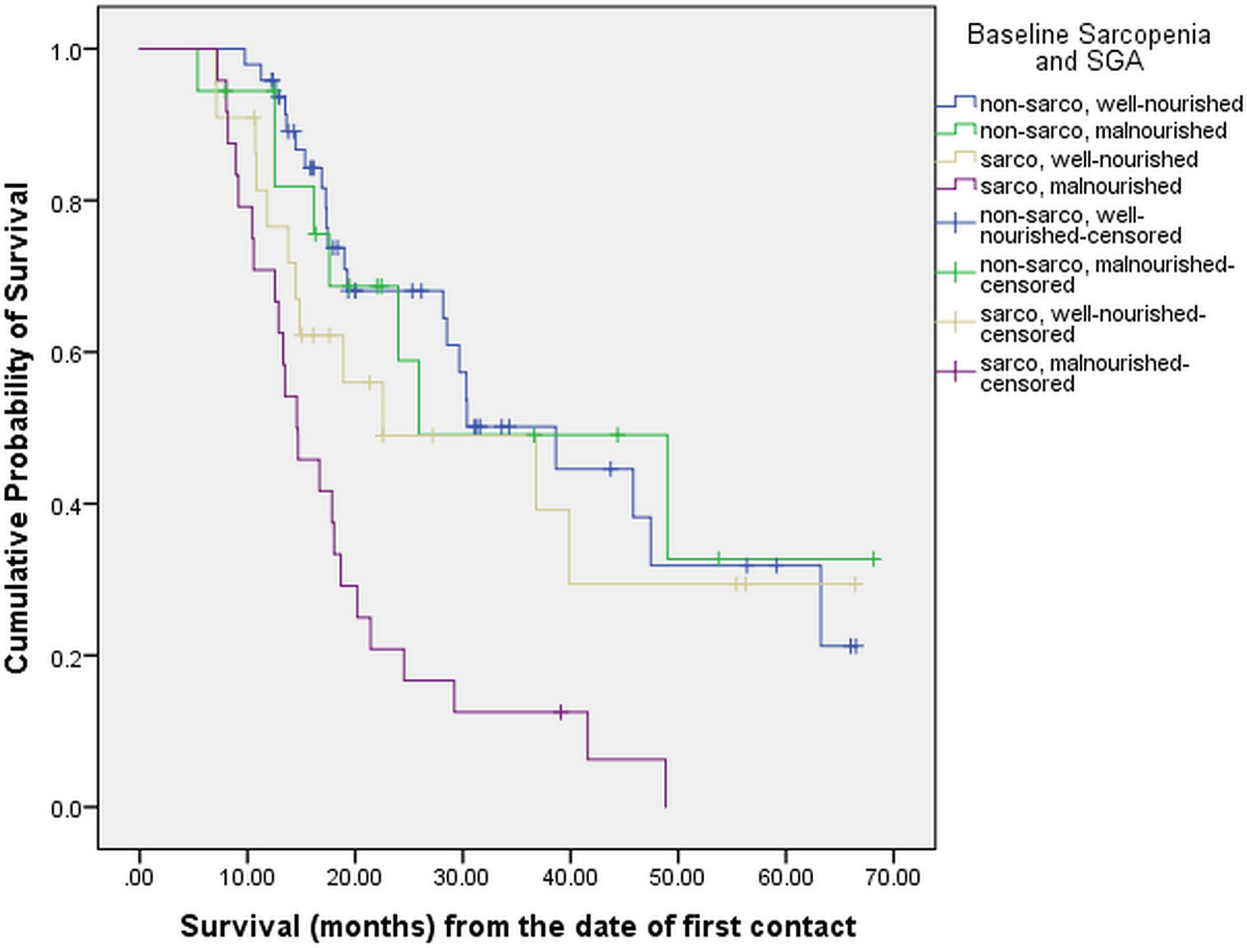
Overall survival stratified by baseline sarcopenia and SGA (N = 112).

On multivariate analysis (**Table 4**), after adjusting for age, gender, tumor stage, BMI, treatment history and SGA, patients with sarcopenia had 3 times greater risk of mortality compared to those without sarcopenia (p = 0.001). No other variable was found to be statistically significant on multivariate analysis. Upon using different combinations of sarcopenia and nutritional status, the median survival of patients with both sarcopenia and malnutrition (n = 24) was 14.6 months (95% CI: 10.5 to 18.6) as compared to the median survival of 25.9 months (95% CI: 7.8 to 44.0) in patients who were either sarcopenic or malnourished but not both (n = 40; p = 0.001), as shown in **Table 5** and **Fig 3**.

**Table 4:** Multivariate survival analysis (N = 112).

Variables	HR	95% CI	P value
Sarcopenia			
No (reference)			
Yes	3.0	1.6–5.6	0.001*
SGA			
Well-nourished (reference)			
Malnourished	1.6	0.94–2.8	0.08
Gender			
Male (reference)			
Female	1.3	0.77–2.2	0.34
Prior treatment history			
Newly-diagnosed (reference)			
Previously-treated	1.5	0.88–2.6	0.13
Stage at Diagnosis			
Non-metastatic (reference)			
Metastatic	1.5	0.80–2.7	0.22
Age (continuous)	0.99	0.96–1.02	0.59
BMI (continuous)	1.04	0.99–1.1	0.12

*P < = 0.05

*SGA* subjective global assessment, *BMI* body mass index, *HR* hazard ratio, *CI* confidence interval

**Table 5:** Median survival based on sarcopenia and nutritional status (N = 112).

Categories (n)	Median Survival in Months	95% CI	P value
Sarcopenic malnourished (n = 24)	14.6	10.5–18.6	<0.001*
Either sarcopenic or malnourished but not both (n = 40)	25.9	7.8–44.0	
- Sarcopenic well-nourished (n = 22)	22.5	7.2–47.1	
- Non-sarcopenic malnourished (n = 18)	26.1	6.8–52.8	
Non-sarcopenic well-nourished (n = 48)	38.6	25.6–51.6	

*P < = 0.05

*CI* confidence interval, *n* sample size

Similar to the stratified univariate analysis reported earlier, we also conducted a multivariate analysis separately for stages I-III and stage IV after adjusting for the same set of variables. For stages I-III patients (n = 46), the presence of sarcopenia was not significantly associated with mortality (HR = 1.6; 95% CI: 0.53–4.9; p = 0.40). However, among stage IV patients (n = 66), those with sarcopenia had 4 times greater risk of mortality as compared to those without sarcopenia (HR = 4.0; 95% CI: 1.7–9.3; p = 0.001).

## Discussion

Although sarcopenia and malnutrition have previously been associated with prognosis in colorectal cancer, all studies conducted to date have addressed these two factors individually. We investigated the association between sarcopenia and overall survival in colorectal cancer after adjusting for the effects of malnutrition. We noted several key findings of interest.

First, patients with sarcopenia were significantly older and had a significantly lower BMI, a finding similar to that reported by Takeda et al. in patients with advanced lower rectal cancer [12], despite the differences in the definitions of sarcopenia used. A meta-analysis of 12 studies in non-metastatic colorectal cancer also reported a relatively lower BMI in sarcopenic patients as compared with those in the non-sarcopenia group [3]. Despite this finding, it is still important to recognize that sarcopenia can be present in patients with high BMI (sarcopenic obesity).

Second, sarcopenia occurred in ~30% of individuals classified as well-nourished by SGA in our study. Similarly, ~45% of individuals classified as malnourished by SGA were non-sarcopenic. This finding is similar to what other authors have reported previously, although in different patient populations. For example, a study by Tandon et al. in 142 patients with cirrhosis listed for liver transplantation reported that sarcopenia was present in 40% of the patients classified as well-nourished by SGA [30]. Similarly, Sheean et al. in their cross-sectional study in 56 patients with respiratory failure, reported sarcopenia to be present in 50–60% of patients ranked as normal nourished [29]. This finding suggests that using SGA alone to evaluate nutritional status in cancer may fail to identify patients with significant skeletal muscle mass loss. Consequently, it is important to assess for sarcopenia in conjunction with SGA in patients with advanced colorectal cancer to obtain a more comprehensive picture of underlying malnutrition and skeletal muscle mass loss.

The third and most significant finding of the current study is that sarcopenia was independently associated with a 3-fold greater risk of mortality after adjusting for the effects of age, gender, tumor stage, BMI, treatment history and SGA. Malnourished status was associated with a 1.6-fold greater increase in mortality as compared to well-nourished status, however, this finding did not attain statistical significance (p = 0.08) in the final multivariate model. Collectively, these findings suggest that sarcopenia may supersede SGA as a predictor of survival in colorectal cancer. Association of sarcopenia with a greater risk of mortality can potentially be explained in multiple ways. Sarcopenia may be reflective of an increased metabolic activity of a tumor biology that is more aggressive, thereby leading to systemic inflammation [14,35]. Studies have also demonstrated a strong association between low skeletal muscle mass and the presence of a systemic inflammatory response, the negative impact of which on cancer outcomes is well documented [36,37]. Furthermore, sarcopenia might be associated with chemotherapy toxicities, which in turn could lead to reduction in chemotherapy doses or termination of chemotherapy altogether [18]. Finally, patients with sarcopenia might also be susceptible to infections leading to adverse outcomes. More work is needed to better delineate the mechanisms through which sarcopenia leads to poor clinical outcomes in cancer.

We also evaluated whether the combined presence of sarcopenia and malnutrition was associated with a greater risk of mortality compared to either sarcopenia alone or malnourished status alone, but not both. Interestingly, we found that co-existence of sarcopenia and malnutrition is associated with significantly worse survival in colorectal cancer compared to just one of those conditions being present. This finding suggests that assessment for sarcopenia and SGA together can provide greater prognostic information as compared to each one of them considered individually.

Colorectal cancer has been extensively examined with reference to CT-derived body composition, and most studies have reported that SMI is associated with survival [2,12–17]. In fact, a recently published systematic review and meta-analysis of 5,337 patients from 12 studies in non-metastatic colorectal cancer demonstrated a significantly decreased overall survival in the sarcopenia group as compared with the non-sarcopenia group (HR = 1.63, 95% CI = 1.24–2.14, p<0.01) [3]. Another meta-analysis based on 7,843 patients with solid tumors (hepatocellular, pancreatobiliary, gastroesophageal, urothelial, renal cell and colorectal) from 38 studies reported that SMI lower than the cut-off (definitions of sarcopenia as defined in the individual studies were used) was associated with poor OS (HR = 1.44, 95% CI = 1.32–1.56, p <0.001) [38]. However, none of these studies have controlled for the effect of malnutrition in their analyses, which by itself has been reported to be an independent negative prognostic factor in colorectal cancer. To the best of our knowledge, ours is the first study to evaluate the prognostic effect of sarcopenia in colorectal cancer after adjusting for the potential confounding effects of malnutrition and other relevant factors.

CT-derived sarcopenia evaluation is very attractive for patients with metastatic cancer. These patients routinely undergo sequential CT scans to evaluate response to therapy. The fact that calculation of sarcopenia score in these patients is relatively inexpensive and does not need much expertise, suggests that adding sarcopenia assessment to conventional nutritional evaluation such as SGA has the potential to identify additional individuals at risk who might benefit from early nutritional intervention. Consequently, the results of our study support the routine measurement of SMI as part of the clinical and nutritional assessment in patients with colorectal cancer. However, further prospective studies with larger sample sizes are needed to validate our findings. Moreover, before the findings of this study can be utilized to influence treatment decisions in the clinic, future research should also evaluate whether enhancing muscle mass and function can improve overall survival, and if so, through what underlying mechanisms.

We acknowledge the limitations of this study. This major limitation is that this is a single-institution retrospective cohort study. Despite conscious effort to monitor and control for them, this study may have been affected by biases inherent in retrospective cohort studies. Our study had a small size, however, the differences in survival across different sarcopenic groups were striking despite the small sample size of 112. Since the patient cohort was limited to only those patients who spoke English, this study sample is not broadly representative of colorectal patients in general. The European Working Group on Sarcopenia in Older People defines sarcopenia as the presence of both low muscle mass and low muscle strength or low physical performance, however, muscle function and strength could not be measured in this study because of its retrospective design [39]. In addition, there is no generally accepted definition of sarcopenia for CT-based measurements and there are no standardized cut-off values determined. As a result, different cut-offs can lead to different results making cross-study comparisons difficult. Another major limitation is that we were not able to control for patient co-morbidities due to lack of relevant data. Given that co-morbidities can potentially influence patient survival, lack of adjustment for them leaves this study at a high risk of confounding. Observer bias can affect SGA assessment since it is a subjective method that relies on the dietitian’s ability to collect and interpret data. In this study, we did not perform an assessment of the interrater reliability of different SGA users. However, the potential for this bias was minimized by restricting the use of the SGA to well-trained dietitians who had an expertise and prior experience with the use of this instrument. We acknowledge that restricting the analysis to newly diagnosed patients (patients with no prior treatment history) would have been more accurate, since it would have allowed for evaluation of true overall survival time i.e. time from the date of diagnosis to the date of death. However, doing so would have caused a significant reduction in the sample size (from 112 to 68). In our study, the survival time was calculated from the day of first visit at our hospital because information on sarcopenia and SGA was not available at the time of diagnosis for previously treated patients. This drawback emphasizes the need for conducting prospective studies having nutritional information available since the date of diagnosis. Also, we included colon and rectal cancers together in the same analysis because of their anatomical proximity and similarity with respect to nutritional challenges. That said, future studies should investigate these two cancers separately given that they have a different disease and treatment trajectory. Finally, the use of various treatment regimens by our patients has the potential to bias the results of the present study. Notwithstanding the study limitations described above, to the best of our knowledge, this is the first preliminary study to report that sarcopenia has a negative impact on long-term survival in patients with colorectal cancer independent of the effects of malnutrition.

## Conclusion

The exploratory study suggests that presence of sarcopenia supersedes the presence of malnutrition as a predictor of survival in colorectal cancer. Co-existence of sarcopenia and malnutrition was associated with worse survival in colorectal cancer compared to just one of those conditions being present. Prospective studies with large sample sizes are needed to confirm these findings.
